# Initial Analysis of Plant Soil for Evidence of Pathogens Associated with a Disease of Seedling *Ocotea monteverdensis*

**DOI:** 10.3390/microorganisms13071682

**Published:** 2025-07-17

**Authors:** William D. Eaton, Debra A. Hamilton, Alexander Lemenze, Patricia Soteropoulos

**Affiliations:** 1 Biology Department, Dyson College, Pace University, New York, NY 10038, USA; 2 Department of Environment and Development, University for Peace, El Rodeo de Mora, San José 10701, Costa Rica; 3 Vermont Cooperative Fish and Wildlife Research Unit, Rubenstein School of the Environment and Natural Resources, University of Vermont, Burlington, VT 05405, USA; debrahamiltonmv@gmail.com; 4 Monteverde Institute, Monteverde, Puntarenas 60109, Costa Rica; 5 Molecular and Genomics Informatics Core (MaGIC), Rutgers New Jersey Medical School, 205 South Orange Avenue, Newark, NJ 07103, USA; alex.lemenze@rutgers.edu; 6 Genomics Center, Rutgers New Jersey Medical School, The State University of New Jersey, 185 South Orange Ave, MSB F653, Newark, NJ 07103, USA; soteropa@njms.rutgers.edu; 7 Department of Microbiology, Biochemistry and Molecular Genetics, Rutgers New Jersey Medical School, The State University of New Jersey, 185 South Orange Ave, MSB F653, Newark, NJ 07103, USA

**Keywords:** tropical seedling disease, fungal plant pathogens, *Mycosphaerella* disease

## Abstract

Seedlings of the ecologically important, critically endangered tree *Ocotea monteverdensisis* experience high mortality in the Monteverde, Costa Rica, cloud forests at the onset of the wet season, yet there are no studies suggesting the disease etiology. Here, healthy and diseased plant root and bulk soils were analyzed for various carbon and nitrogen (N) metrics and respiration levels, and DNA sequence-based bacterial and fungal community compositions. All nitrogen metric levels were greater in diseased vs. healthy plant root soils, which could enhance pathogen growth and pathogenic mechanisms. Greater DNA percentages from several potential pathogens were found in diseased vs. healthy plant root soils, suggesting this disease may be associated with a root pathogen. The DNA of the fungus *Mycosphaerella* was at greater levels in diseased vs. healthy plant root soils than other potential pathogens. *Mycosphaerella* causes similar diseases in other plants, including coffee, after onset of the wet season. The *O. monteverdensis* disease also occurs in seedlings planted within or near former coffee plantations at wet season onset. Distance-based linear model analyses indicated that NO_3_^−^ levels best predicted the pattern of fungal pathogens in the soils, and *Mycosphaerella* and *Tremella* best predicted the patterns of the different N metrics in the soils, supporting their possible roles in this disease.

## 1. Introduction

Restoration of tropical forests is essential, and the survival of seedlings is critical to the success of these efforts. Multiple factors may impact seedling survival, with fungal pathogens shown to be a significant, if not the greatest, threat to seedling survival [1]. Recent reports indicate that potential fungal pathogens are increasing in response to ongoing climate change, with expanding species ranges, greater diversity, and enhanced resilience [2]. It is believed that natural systems may be more vulnerable to increasing pathogens [3], and the warm, humid tropics may be especially susceptible. In light of these growing concerns, emerging models are beginning to reveal the complexity of these impacts [4]. Given the known challenges and costs associated with tropical forest restoration, identifying and protecting plants from these fungal pathogens is a priority. A crucial first step is to identify the specific types of fungal pathogens harming these restoration efforts.

A severely threatened tree species, *Ocotea monteverdensis*, is suspected of suffering high mortality rates due to a systemic pathogen that suddenly causes necrosis of seedling leaves and subsequent death with the onset of rains during the wet season. It is one of approximately 96 species of the Lauraceae family that comprises a large percentage of the diversity of the forests in the Monteverde region of Costa Rica. This tree is listed as globally “critically endangered” [5,6], as less than 1000 mature individuals remain within its principal occupancy range in Monteverde, where a large percentage of the mature forest of this region was cleared for timber, human development, or agricultural uses [6]. Only 5% of the remaining trees in this region are on permanently protected land [7].

Due to the highly threatened status of *O. monteverdensis* and its ecological importance as the preferred food source of large frugivorous birds such as the vulnerable three-wattled bellbird (*Procnias tricarunculatus*) [8,9], there have been multiple coordinated efforts to protect mature trees and plant seedlings and increase awareness by the Fundacion Conservacionista Costarricense (FCC) and the Monteverde Institute. The emphasis of these programs has been to augment existing forests and restore abandoned pastures with plantings of threatened tree species, such as *O. monteverdensis*, and to specifically advance the knowledge regarding the factors impacting the survival of *O. monteverdensis*.

There has been a limited number of studies concerning *O. monteverdensis*, even though its ecological importance in cloud and humid forests has been recognized [6,8,10]. The genetic variation within the limited population size is not known, and reproductive success to mature tree stage has not been determined. There are indications that *O. monteverdensis* is not regenerating its population sufficiently, which is thought to be due to decreased amounts of habitat appropriate for regeneration [11,12,13].

Collaborative research with the FCC restoration program found high mortality rates of *O. monteverdensis* seedlings planted in both forest fragments and abandoned pasture habitats. The seedling mortality continues past its fourth year post-planting, instead of reaching a plateau after two years, as found with other tree species [13,14]. Of special note is the observed sudden death of seedlings that starts with necrosis of most leaves shortly after the first rains of the May to June rainy season. Additional studies examining natural regeneration of this tree species have shown concerns regarding seedling success [13]. To date, even with this suspicion of pathogen involvement in seedling mortality, no work has been conducted on the root soil microbial community or activity, nor on the carbon and nitrogen components associated with healthy and diseased seedlings.

The current study was conducted to address the potential role of a pathogen in the mortality of young *O. monteverdensis*. While necrotrophic fungal pathogens are more common [15], the sudden, systemic death of *O. monteverdensis* seedlings suggests a soil-inhabiting pathogen. For this work, the soil carbon and nitrogen metrics were assessed, and the overall DNA sequence-based bacterial and fungal community compositions were determined within the root-associated and the plant bulk soil (soil within the dripline) of seedlings planted in reforested areas within the Monteverde premontane wet forest. In addition, the most common fungal and bacterial genera from each sample were determined, with any potential pathogens identified.

## 2. Materials and Methods

### 2.1. Sample Site and Collections

The Fundacion Conservacionista Costarricense (FCC, www.fccmonteverde.org) has made significant conservation contributions by distributing and planting over 300,000 seedlings from 140 native tree species on the property of nearly 500 landowners throughout the Bellbird Biological Corridor on the Pacific slope of Costa Rica. This has included the planting of over 5000 *O. monteverdensis* seedlings, with the monitoring of 950 individuals.

*O. monteverdensis* is a critically endangered tree that is endemic to Costa Rica, with its core population found in the tropical premontane wet life zone on the Pacific slope of the Cordillera de Tilarán (between 10°25′ N and 10°16′ N, and longitudes 84°42′ W and 84°55′ W) at elevations between 1200 and 1500 m asl [9,14,16,17]. *O. monteverdensis* is a large canopy tree that produces 3–4 cm-long avocado fruits every three years on average, with 64% synchrony within the species [8]. Once common in the Monteverde region, it was previously harvested for its fine red wood [9].

**Figure 1:**
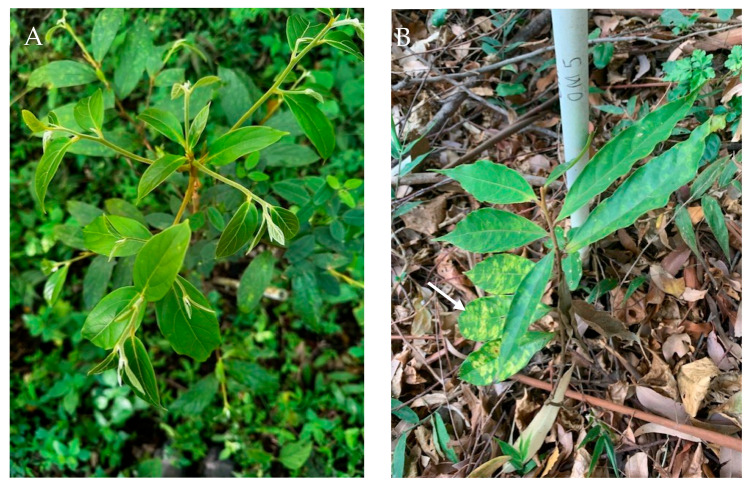

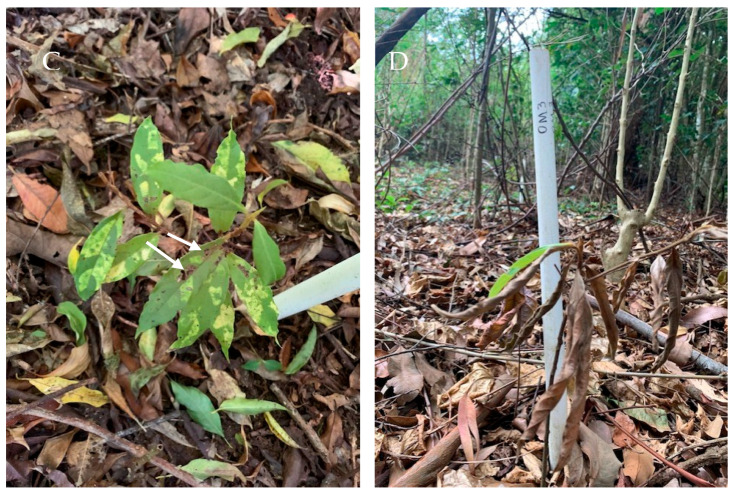
*Ocotea monteverdensis* seedlings at different stages of disease. (**A**) Non-diseased, healthy seedling; (**B**) seedling demonstrating early stages of chlorosis (arrow); (**C**) seedling demonstrating later stages of chlorosis and brown spots (arrows); (**D**) seedling dying with curled leaves.

There is a suspicion that the high mortality of the seedlings within the first planted year may be caused by a pathogen. This is based on the observation that the seedling mortality post-planting is associated with a sequential pattern of chlorosis (Figure 1A), followed by development of brown spots (Figure 1B,C), then curling and wilting of the leaves (Figure 1D), followed by a sudden death and dropping of all its leaves, leading to plant death. This usually occurs after the start of the rainy season and involves seedlings that are in their first two years after planting.

The site used for this study is located at 1430 m asl in the premontane wet life zone and receives an average of over 2863.2 mm of precipitation per year [18]. In 2021, 6 seedlings of diseased and 6 of non-diseased *O. monteverdensis* were used for this study. The seedlings were raised from the same seed stock, in the same nursery with equivalent watering and seedling care, and with the same soil source used in the seedling bags. Healthy seedlings were planted in 2020 at the FCC’s Crandell Memorial Reserve, augmenting a reforestation effort of abandoned pasture done in 2008, and all were planted under 13-year-old *O. monteverdensis*, thereby resulting in light availability being reduced to a partial shade status. In addition, this method ensured all trees were planted in soil that had experienced the same land-use history, resulting in similar soil characteristics throughout the reforested area at the time of seedling planting. Of the 82 planted seedlings, 62 had survived at the time of sampling in 2021. They were planted with an average height of 20.3 cm with 13.8 leaves. After one year, the average height was 23.8 cm with 16.7 leaves. The 6 healthy and 6 diseased young *O. monteverdensis* used in this study were all 20–30 cm in height and separated from adjacent sampled seedlings by >2 m, had been propagated from seeds in the FCC nursery and maintained there for about 1 year, and had been planted in the study area 1 year prior to sampling.

For collection of soil from the 6 heathy and 6 diseased plants, 4 soil cores 10 cm deep were collected at the cardinal points around the plant at a distance from the plant equal to 75% of the dripline distance (between 10 cm and 16 cm from the plant base) using a 7.5 cm × 15 cm × 1.25 cm soil profiler. The 4 cores from individual plants were stored in sterile plastic bags. These samples are hereafter called the plant bulk soil samples. After collection of the soil from an individual plant, cuts were made in the soil around the same plant that were approximately a 3 cm-diameter distance from the plant base to allow easy removal of the plant and root system. This plant root soil sample was placed in a sterile bag. In the lab, the loose soil and the soil attached to the roots were collected and stored in sterile bags, and hereafter are called the plant root soil samples. This process resulted in the collection of 6 healthy and 6 diseased plant bulk soil and plant root soil samples to be used for analysis. All collection tools and gloves were disinfected with 70% ethanol between individual plants and between the collection of plant soil and plant root samples to inhibit cross-contamination. All field moist soil samples were passed through an ASTM No. 4 sieve (about 5 mm openings) previously disinfected with 70% ethanol.

### 2.2. Soil Environmental Metrics

Subsamples from each sample were analyzed at the Center for Tropical Agriculture Research and Education (CATIE) Laboratory in Turrialba, Costa Rica, for all C and N metrics. This included determination of the total organic C (TOC) mass by dry combustion analysis [19], the total N (TN) mass by the standard Kjeldahl method using a Thermofinigan EA12 autoanalyzer (San Jose, CA, USA), the respiration level as CO_2_ (Resp) by standard closed-vessel methods [20], and the levels of both nitrate (NO_3_^−^) and ammonium (NH_4_^+^) from 2 M KCl extracts using the spectrophotometric methods previously described [20].

### 2.3. DNA Extraction, Sequencing, and Bioinformatics

Total soil DNA was extracted from three 0.33 g replicate sub-samples from each soil sample using the MoBio PowerSoil DNA Isolation Kit (MO BIO Laboratories Inc., Carlsbad, CA, USA), with the concentration and purity determined as the A_260_/A_280_ ratio of the individual DNA samples using a NanoDrop 1000 spectrophotometer (ThermoFisher Scientific, Waltham, MA, USA). The PCR amplification of DNA targeting the nuclear internal transcribed spacer (ITS) ribosomal RNA gene region of fungi [21] and the v3 and v4 of 16S ribosomal RNA gene region of bacteria [22] are now commonly used to identify microbial taxa. For the current project, previously described methods were used [23] in a 2-step PCR amplification process that included use of the specific PCR primer set 16Sv3F and 16Sv4R [24] for bacterial 16S rRNA amplification, and the primer set ITS4 and ITS1F [25] for fungal ITS region amplification. The PCR steps were the same for use of the 16S and the ITS primer sets. The first step of PCR amplification was performed on 2 µL of sample DNA in a reaction buffer containing 200 mM of Tris-HCl, 500 mM of KCl, a pH of 8.4, 50 mM of MgCl_2_, 10 mM of dNTPs mix (10 mM), 10 mM of forward primer, 10 mM of reverse primer, and 5 U/µL of Invitrogen Platinum Taq polymerase (5 U/µL). The PCR conditions were 95 °C for 5 min; 35 cycles of 94 °C for 40 s, 46 °C for 1 min, 72 °C for 30 s, and 72 °C for 5 min; and held at 4 °C. The second PCR step was performed using the purified 1st PCR product as a template with Illumina adapter tailed specific target primers, following the same protocol as mentioned before, except that 30 cycles were used. All raw DNA sequence reads were assessed for quality control (FASTQC v 0.12.0), then trimmed and filtered for quality (Cutadapt v 3.2). The 16S amplicon sequence variants that passed the quality filter were assigned taxonomic identification (DADA2 v 1.20.0) from phylum to genus based on the SILVA rRNA database (build 138) for bacteria. The fungal ITS amplicon sequence variants that passed the quality filter were taxonomically identified from phylum to genus using the Ribosomal Database Project Classifier (https://github.com/rdpstaff/classifier, accessed on 28 October 2021) with the UNITE database for fungi [26]. All DNA sequence data were submitted to the NCBI Sequence Read Archive (NCBI Bioproject numbers PRJNA1019742 and PRJNA646467). To normalize the effects of uneven numbers of sequences per sample, the number of times a specifically identified genus appeared in a sample was converted to mean proportion of the sequences (MPS) per sample, as previously recommended [27].

The total of all bacterial and fungal taxa identified by these DNA sequencing methods are hereafter referred to as the total bacterial and the total fungal communities in this study. A variety of resources was used to identify bacterial and fungal genera that were potential plant pathogens, and also to determine other potential functional groups of various bacterial and fungal genera (Table S1). The bacterial and fungal genera that were identified as potential plant pathogens are hereafter referred to as bacterial and fungal pathogen communities.

### 2.4. Data Analysis

All differences in the mean values of the soil sample TOC, TN, NH_4_^+^, NO_3_^−^, and respiration (CO_2_) were conducted using one-way ANOVA tests in SPSS (v.27, Armonk, NY, USA), with the sample year as the predictor variable. Prior to ANOVA, Levene’s test was performed in SPSS to determine homogeneity of the variances of the data. All data had *p* values > 0.05, suggesting that the use of the Tukey’s HSD post-hoc test was appropriate.

The MPS values for all microbial MPS values were 4th-root transformed, as recommended by Ref. [28]. The 4th-root-transformed MPS values were converted into Bray–Curtis similarity matrices and analyzed for differences in the composition of the total bacterial, bacterial pathogenic, total fungal, and fungal pathogenic communities using the analysis of similarity (ANOSIM) routine performed in PRIMER-E v.6 and PERMANOVA+ (Plymouth, UK) [28], which provided R and *p* values for the main and pairwise tests. The strength of any of these differences was determined using the Canonical Analysis of Principal Coordinates (CAP) method [29,30] in PRIMER-E with the PERMANOVA + add-on. The CAP axis R^2^ values ≥ 0.7 represent strong compositional differences, R^2^ = 0.5 to 0.69 represents moderate differences, R^2^ = 0.20 to <0.5 represents weak differences, and R^2^ < 0.20 suggests no differences. The differences in MPS values of the bacterial pathogens and fungal pathogens between the healthy and diseased plant bulk soils and plant root soils were determined using the Mann–Whitney U test in SPSS.

To further compare differences in microbial community composition between the different healthy and diseased plant soil samples, the Similarity Percentage (SIMPER) analysis in PRIMER-E routine was used on the 4th-root-transformed MPS data to demonstrate differences in the percent taxonomic dissimilarity. In addition, the richness (as number of genera) and diversity (as Shannon’s H index) were calculated, and differences in mean values of these metrics between the healthy and diseased bulk soil and root soil samples were determined using the Mann–Whitney U test in SPSS. Lastly, the differences in MPS of the more common (MPS > 1%) total fungal and total bacterial taxa were compared for the different paired healthy and diseased soil samples by Mann–Whitney analyses in SPSS. In addition, the potential functions of these taxa were determined using the resources mentioned above.

Distance-based linear model (DistLM) analyses in Primer E v.6 and PERMANOVA+ were used to analyze the relationships between the microbial community compositions and the soil environmental parameters in both healthy and diseased plant bulk and root soils [28,30]. Tests were conducted to determine the influence the environmental metrics had on the different microbial communities and the influence the communities had on the environmental metrics. In both cases, log (x + 1) environmental metrics and the 4th-root-transformed MPS values were used. For these analyses, a stepwise selection process was used, along with an AICc (Akaike Information Criterion Corrected) selection criterion, which is recommended for small datasets, such as the ones from this study, in which the number of samples (N) is small relative to the number (v) of predictor variables, such that N/v < 40 [30]. In all cases, 9999 permutations were used as recommended [28,30].

## 3. Results

### 3.1. Differences in Soil Carbon and Nitrogen Metrics

There were no differences (all *p* values > 0.09) in the levels of TOC or respiration between the diseased compared to the healthy plant root soils, but the levels of TN, NH_4_^+^, and NO_3_^−^ were all greater (*p* range = 0.046 to 0.055) in the diseased plant root soils compared to those in the healthy plant root soils (Table 1). The levels of TN, NH_4_^+^, NO_3_^−^, and TOC were all greater (all *p* values < 0.001) in the diseased plant bulk soil compared to those in the healthy plant bulk soil, but there were no differences in respiration levels between the soils (Table 1).

### 3.2. Differences in Community Compositions

The ANOSIM results (Table 2) showed there were differences in the total bacterial community compositions between the healthy and diseased plant root (R = 0.566, *p* = 0.003) and plant bulk soils (R = 0.416, *p* = 0.0002). Similarly, there were differences in bacterial pathogen communities between the healthy and diseased plant root (R = 0.563, *p* = 0.004) and plant bulk soils (R = 0.485, *p* = 0.002). The CAP findings for these comparisons (Table 2) showed that all these differences were considered strong differences in community composition (CAP R^2^ range of 0.706 to 0.876, *p* values = 0.0001 and 0.0002). The ANOSIM results showed there were differences in the total fungal and fungal pathogen community compositions (Table 2) between the healthy and diseased plant root soils (R = 0.752, *p* = 0.004; and R = 0.554, *p* = 0.002). There were also differences (Table 2) in the fungal pathogen community composition between the healthy and diseased plant bulk soil (R = 0.231, *p* = 0.05), but not in the total fungal community composition between the healthy and diseased plant bulk soil (R = 0.046; *p* = 0.273). The CAP findings for these comparisons (Table 2) showed that the differences in the total fungal and fungal pathogen community compositions were strong between both the healthy and diseased plant root soils and the plant bulk soils (R^2^ = 0.801, *p* = 0.0022, and R^2^ = 0.855, *p* = 0.0008). The CAP results showed that between the healthy and diseased plant bulk soils the differences in the total fungal community compositions were very weak (R^2^ = 0.223; *p* = 0.0022) and the differences in community composition of the fungal pathogens were moderate (R^2^ = 0.599; *p* = 0.008).

**Figure 2:**
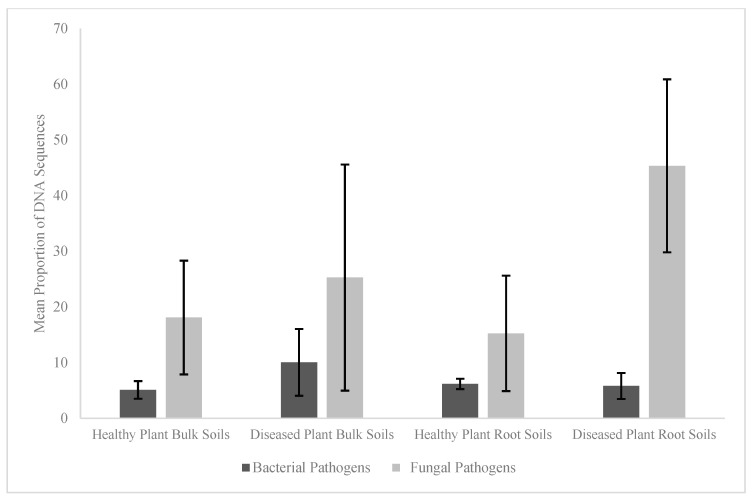
Differences in the mean proportion of DNA sequences (MPS) of the potential bacterial pathogens and fungal pathogens (identified as described in the Section 2.3) in the plant root soils and plant bulk soils of diseased and healthy *Ocotea monteverdensis* planted in a Costa Rican primary forest for 1 year.

There were no differences observed in the MPS (Figure 2) of the bacterial pathogen community between the healthy and diseased plant root soils (*p* = 0.873) or plant bulk soils (*p* = 0.109). There was also no difference in the MPS of the fungal pathogens between the healthy and diseased plant bulk soils (*p* = 0.749). However, the MPS of the fungal pathogens was greater (Figure 2) in the diseased plant root soils compared to the healthy plant root soils (45.35% vs. 15.25%; *p* = 0.016).

**Figure 3:**
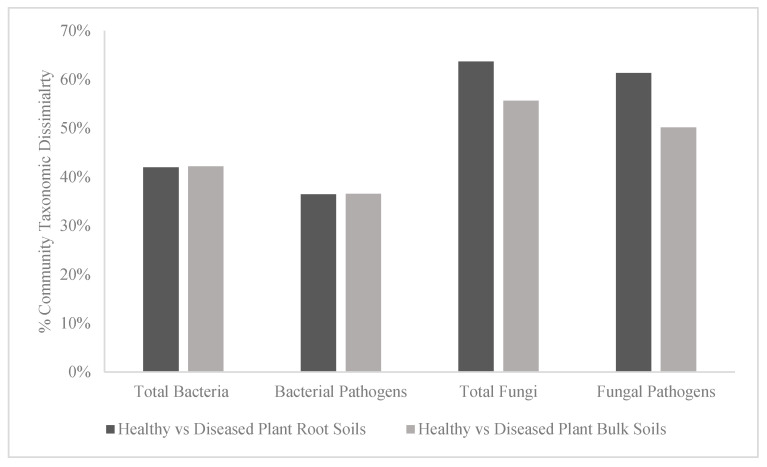
Results of the Similarity Percentage (SIMPER) analyses comparing the % taxonomic dissimilarity of the communities of total bacteria, bacterial pathogens, total fungi, and fungal pathogens (identified as described in the Section 2.3) in plant root soils and plant bulk soils of diseased and healthy *Ocotea monteverdensis* planted in a Costa Rican primary forest for 1 year.

There were no differences in percent taxonomic dissimilarity (Figure 3) of the total bacterial or bacterial pathogen communities between the healthy and diseased plant bulk soil or the healthy and diseased plant root soil (41.97% vs. 42.19% and 36.42% vs. 36.52%, respectively). However, there were differences in the percent taxonomic dissimilarity (Figure 3) of the total fungal and fungal pathogen communities between the healthy and diseased plant root soils (55.65% vs. 63.66% and 50.16% vs. 61.34%, respectively). There were no differences in the richness or diversity of the total bacterial or bacterial pathogen communities (Table 3) between the healthy and diseased plant roots or plant bulk soils (all *p* values > 0.4). There were also no differences in the richness or diversity of the total fungal communities (Table 3) between the healthy and diseased plant bulk soils (*p* values > 0.26). However, the richness of the total fungal and the fungal pathogen communities was greater in the diseased compared to the healthy plant root soils (86.83 vs. 23.51, *p* = 0.0002; and 20.17 vs. 8.51, *p* = 0.004). Similarly, the diversity of the total fungal and the fungal pathogen communities was greater in the diseased compared to the healthy plant root soils (4.26 vs. 2.74, *p* = 0.0003; and 2.77 vs. 1.76, *p* = 0.005).

There were many differences in the MPS of specific bacterial genera (with MPS levels > 1%) between the healthy and diseased plant bulk soils (Table 4); however, *Clostridum* was the only potential bacterial plant pathogen [31] with greater MPS levels in the diseased compared to the healthy plant bulk soils (3.50% vs. 0.74, *p* = 0.001). There were few differences in the MPS of bacteria with an MPS > 1% in either the healthy or diseased plant root soils, although *Clostridium* was present at greater MPS levels in the diseased compared to the healthy plant root soils (3.18% vs. 0.36%, *p* = 0.019).

There were eight fungal genera with MPS levels > 1% in either the diseased or healthy plant bulk soils (Table 5), but the plant pathogen *Ilyonectria* was the only one of these genera with MPS levels that were different between the different plant bulk soils, with a greater MPS in the healthy compared to the diseased plant bulk soils (8.68% vs. 1.28%, *p* = 0.0039). There were 10 fungal genera with an MPS > 1% that were present in either the diseased or the healthy plant root soils (Table 5). The complex organic C compound-degrading (CCD) genus *Apiotrichum* was present at greater MPS levels in the healthy compared to the diseased plant roots (62.07% vs. 26.58%, *p* = 0.0163), while the CCD genus *Saitozyma* and the arbuscular mycorrhizal genus *Glomus* had MPS levels that were moderately (not statistically significantly) greater in the roots from healthy compared to diseased plants (9.74% vs. 4.17%, *p* = 0.0781; and 3.00% vs. 1.48%, *p* = 0.1093, respectively). Seven of the 10 genera had greater MPS values in the diseased compared to the healthy plant root soils. The plant pathogen *Mycoshpaerella* was present in the diseased plant root soils at a much greater MPS level than in the healthy plant root soils (11.65% vs. 3.20%, *p* = 0.0039), as were the plant pathogens *Dipodascus*, *Didymella*, and *Acrocalymma*, but at much lower MPS levels (1.97% to 2.76% vs. 0.01% to 0.37%, *p* = 0.0021 to 0.0225). There were two other genera worth noting. The MPS of the fungal Oomycete and algal parasite *Rozella* had a much greater MPS level in the diseased compared to the healthy plant root soils (13.3% vs. 0.65%, *p* = 0.0038). In addition, the MPS of the plant root-associated fungal genus *Archaeorhizomyces* also had much greater MPS levels in the diseased compared to the healthy plant root soils (11.26% vs. 0.23%, *p* = 0.0033).

**Figure 4:**
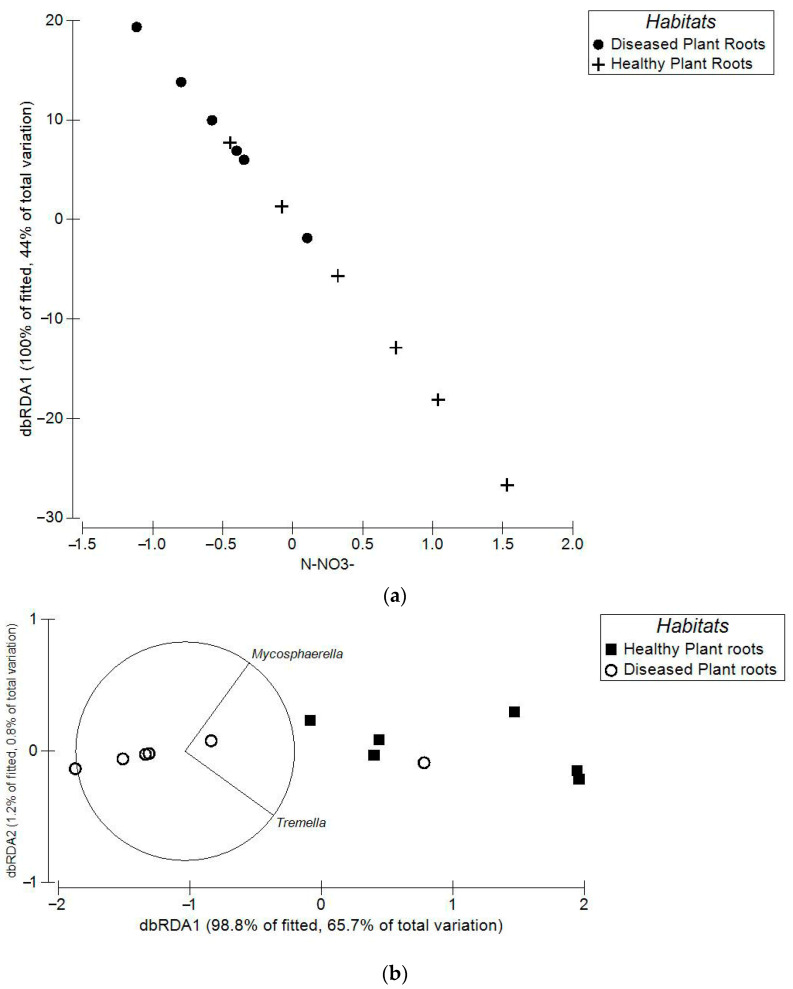
The results of the DistLM analyses determining the relationships between the microbial community compositions and the soil environmental parameters in the healthy and diseased plant root soils. (**a**) The influence of environmental metrics on the fungal pathogen communities in the two soils. (**b**) The influence of the fungal pathogen communities on the environmental metrics in the two soils.

The DistLM results (Figure 4a,b) showed there were no statistically valid relationships between the bacterial pathogen community and the environmental metrics in any habitats or in the fungal pathogen community in the healthy or diseased plant bulk soils. However, there were statistically valid relationships between the fungal pathogen community and some of the environmental metrics in the diseased and healthy plant root soils. Specifically, the levels of NO_3_^−^ influenced 43.96% of the fungal pathogen community compositions in the diseased and healthy plant root soils (Figure 4a, AICc = 69.96, pseudo-F = 7.84, *p* = 0.0037), and the MPS levels of *Mycosphaerella* and *Tremella* combined greatly influenced 66.52% of the levels of TN, NO_3_^−^, and NH_4_^+^ in the diseased and healthy plant root soils (Figure 4b, AICc = 8.38, pseudo-F = 16.11, *p* = 0.002).

## 4. Discussion

The levels of the three nitrogen environmental metrics were all greater in the diseased plant root soils compared to the healthy plant root soils. In addition, the nitrogen metric values and the TOC were greater in the diseased plant bulk soils compared to the healthy plant bulk soils. Studies have shown that increases in the soil N and in either of the two forms of inorganic N can increase the risk of pathogens, causing diseases in plants through several mechanisms. Excess N can enhance the microbial pathogen’s metabolism by causing an increase in production of amino acids and gamma amino butyric acid, which are used by the microbe to enhance its growth rate and pathogenic processes, and also to increase the production and release of various microbial virulence factors [32]. Excess N also is known to decrease the thickness of plant cuticle and wax layers and reduce the amount of lignin produced by a plant, all of which enhance a pathogen’s ability to penetrate the plant surface layers [33]. Furthermore, excess N can inhibit production of plant metabolites that can be protective against pathogens, such as phytoalexins, antimicrobial proteins, amino acids, organic acids, and various defense-related enzymes [33]. It is possible that the greater levels of N in the diseased plant soils could be a result of the decomposition of the dead and dying plant roots. Regardless, given the potential pathogen-enhancing effects of excess N on plants and the greater levels of N found in the diseased plant roots, it is possible that high levels of TN and forms of inorganic N could be enhancing the pathogenesis of a yet unknown pathogen of *O. monteverdensis*.

There were clear differences in total bacterial and the bacterial pathogen community compositions between the heathy and diseased plant roots and plant soils. This suggests that no matter which pathogenic processes are occurring in association with the diseased *O. monteverdensis*, they appear to be influencing the overall bacterial community compositions. However, the greatest differences in composition found for both bacterial groups were between the healthy and diseased plant root soils. This suggests that the disease has a greater influence on the bacterial community near the roots, providing support that the disease is likely root-associated. That said, the only potential plant pathogen found in greater MPS levels in the diseased vs. healthy plant roots or plant bulk soil was *Clostridium*, which was found at MPS levels of about 3–3.5% in the soil and roots of diseased *O. monteverdensis*, but only at about 0.4–0.75% in the healthy plant soil and roots.

There have been six species of *Clostridium* recently identified from the roots and soil of kiwi plants (*Actinidia chinensis* var. *deliciosa* “Hayward”) with a withering and wilting disease, somewhat similar to what has been observed in *O. monteverdensis* [31]. It is interesting that the disease in kiwi plants was enhanced with heavy moisture, which is consistent with observations by the reforestation biologists in the FCC in Costa Rica, who have found that the disease in *O. monteverdensis* seems to occur in the rainy season. Given that *Clostridium* was the only potential bacterial pathogen found in greater levels in the diseased plants than in the healthy plants, it is the only possible bacterial candidate from this study that could be the causative agent for this *O. monteverdensis* disease.

There was also clear evidence of differences in the overall total fungal and fungal pathogen community compositions between the heathy and diseased plants. This indicates that the pathogenic processes associated with *O. monteverdensis* disease are influencing the soil fungal community compositions. Furthermore, as the greatest differences in community composition for both groups was between the healthy and diseased plant root soils, and there were minimal to no differences in these communities between the healthy and diseased plant bulk soils, this strongly supports the idea that this disease of *O. monteverdensis* is linked to a root-associated pathogen.

The known fungal pathogens *Dipodascus*, *Didymella*, *Acrocalymma*, and *Mycosphaerella* all had significantly greater MPS levels in the diseased plant root soils than in the healthy plant root soils, which were also greater than in the healthy or diseased plant bulk soils. *Mycosphaerella* is the fungus perhaps of greatest interest in this study, as its DNA was present in the diseased plant root soils at MPS levels far greater than those of the other pathogens or in the healthy plant root soils. *Mycosphaerella* is considered a plant pathogen; is very common, with over 10,000 species within the genus; and has members of the genus causing plant diseases involving chlorosis, which leads to leaf spot browning, leaf loss, and plant death [34,35] and also has been associated with the *O. monteverdensis* disease. It is also common for *Mycosphaerella* infection and disease to become enhanced during or following periods of heavy rain, which are thought to enhance spore growth and result in easier entry into plant stomata [36,37,38,39]. This pattern of enhanced disease after heavy rain has been observed with *O. monteverdensis* in the Monteverde area. *Mycosphaerella coffeicola* (the asexual form of *Cercospora coffeicola*) is a common pathogen of coffee, causing a leaf spot disease that begins as chlorotic spots on the leaf surface that develop into brown and necrotic lesions. These lesions become discolored with a lighter center and yellow halo, and sometimes fuse into large irregular areas, resulting in the leaves dropping [40,41]. These symptoms in plants have also been observed with the *O. monteverdensis* disease. In addition, similar to the disease occurrence in *O. monteverdensis*, the disease due to *M. coffeicola* in coffee plantations is enhanced after continual wetness for 36–72 h [41]. Lastly, it is also of interest that many of the diseased *O. monteverdensis* plants are seedlings that were planted in areas that had previously been forests before being cleared and used as coffee plantations, with some areas even still containing some abandoned coffee plants within the planted areas. It is certainly premature to suggest that *Mycosphaerella* is the cause of the *O. monteverdensis* disease, but it gives us a target to look for in future studies.

The DistLM showed that the levels of soil NO_3_^−^ had the greatest influence on the fungal pathogen community compositions in the plant root soils, and that the MPS levels of *Mycosphaerella* and *Tremella* combined had the greatest influence on the levels of TN, NO_3_^−^, and NH_4_^+^ in the plant root soils. These results support the concepts of *Mycosphaerella* possibly being an etiologic agent of the disease and of inorganic N levels potentially also playing a role in the disease pathogenesis.

There were two other fungi of interest that warrant further mention. *Archaeorhizomyces* DNA was present at MPS levels much greater in the diseased plant root soils than in the healthy plant root soils. There is little known of this fungus, but it is known as a root-associated fungus that is involved in the decomposition of various plant materials, detritus, and debris found in the root zone, particularly those with low pH and high nutrient turnover, as is associated with diseased plants [42,43,44,45,46]. *Archaerorhyzomyces* is thought to be a decomposer of glucose, cellulose, and other carbohydrates released from dying plants into the root zone [47]. It has been suggested that *Archaeorhizomyces* may be replacing the ectomycorrhizal fungi in the plant roots during above-ground plant senescence and associated root decay, and may be involved in the decomposition of the dead and dying root material released during these periods of plant senescence [43,48,49]. This information and the more than 60× greater MPS levels of *Archaeorhizomyces* in the diseased plant roots suggests that while not the primary pathogen of the *O. monteverdensis* disease, it may be an indicator of the effects this disease has on the *O. monteverdensis* rhizosphere soil microbial community.

The other interesting fungal genus worth noting is *Rozella*, as the DNA of this fungus was present in the diseased plant root soils at much greater MPS levels in the healthy plant root soils. *Rozella* is an endoparasite of the Oomycota; the fungal phyla Blastocladiomycota, Monoblepharidomycota, Chytridiomycota, and Basidiomycota; and the green algal phylum Charophyta [50]. Very little is known of this fungus; however, it has been found to parasitize fungi within soils where there is an increase in the levels of decomposition of plant debris and detritus [51,52]. As such, it is thought to somehow be involved in the decomposition of dead plant roots and other rhizosphere plant debris, although the mechanisms are not known. Nonetheless, this activity could explain its increased abundance in the plant root soils of diseased *O. monteverdensis* compared to the levels in the plant bulk soil of these diseased plants. Thus, the increased *Rozella* abundance, like that of *Archaeorhizomyces*, could be a consequence or indicator of the disease in *O. monteverdensis* seedlings.

## 5. Conclusions

It is unclear what is causing the disease observed in *O. monteverdensis* in the Monteverde cloud forest region of Costa Rica. The evidence from this study strongly points to a root-associated pathogen, based on the greater difference in community composition of the different microbial groups being found between the diseased vs. healthy plant root soils. Although not definitive proof of the etiology of the disease, the data from this work on the fungus *Mycosphaerella* do provide the best evidence for a possible causative agent of the *O. monteverdensis* disease, as it was present in much greater levels in the diseased plant root samples than any other fungal pathogens and has members of the genus causing diseases in plants with similar symptoms to those of the *O. monteverdensis* disease, many of which are more common during or immediately after extremely wet periods, which is also the case with the *O. monteverdensis* disease. Furthermore, this is consistent with *M. coffeicola*, which causes a disease in coffee plants with symptoms similar to those observed with the *O. monteverdensis* disease, which develops after a 36–72 h period of extensive wetness. However, proof of the cause of this disease will require pure culture of a fungal taxa and replication of the disease in young *O. monteverdensis*. This work is planned for the future. While *Mycosphaerella* certainly may not be the causative agent of the disease, it is an interesting possibility that warrants further work. Also, the non-pathogenic fungi *Archaeorhizomyces* and *Rozella* may represent two genera that can serve as secondary indicators of the *O. monteverdensis* disease. It is also possible that the elevated levels of N may be enhancing the growth and/or pathogenic mechanisms of a potential pathogen or are reducing the effectiveness of some of the plant defense mechanisms, both of which could play a role in increasing the prevalence and/or pathogenic outcomes of a microbe associated with this plant disease in *O. monteverdensis*. Ongoing studies include the development of pure fungal cultures from the roots of diseased plants, the determination of the taxonomic identity of these cultures, and the use of these pure cultures in transmission studies with young *O. monteverdensis* seedlings to fulfill Koch’s postulates to determine the etiology of this disease.

## Figures and Tables

**Table 1:** Mean levels of environmental variables in diseased and healthy plant root soils and diseased and healthy plant bulk soils from *Ocotea monteverdensis* planted in a Costa Rican primary forest for 1 year.

	Diseased Plant	Healthy Plant	Mann–Whitney
Metric	Root Soil	Root Soil	*p* Value
TN	58.13 ± 11.27	44.03 ± 11.18	0.052
TOC	763.5 ± 184.78	665 ± 98.56	0.093
NH_4_^+^	6.12 ± 3.01	2.91 ± 2.04	0.055
NO_3_^−^	60.19 ± 27.62	29.25 ± 7.92	0.046
Resp	196.50 ± 71.66	207.53 ± 28.82	0.735
pH	5.92 ± 0.24	5.72 ± 0.15	0.282
% Sand	69.28 ± 4.85	69.26 ± 2.48	0.998
% Silt	21.20 ± 3.92	20.15 ± 2.06	0.951
% Clay	9.51 ± 1.32	10.58 ± 1.02	0.43
BD	2.5 ± 0.6	2.6 ± 0.9	0.761
	**Diseased Plant**	**Healthy Plant**	**Mann–** **Whitney**
**Metric**	**Bulk Soil**	**Bulk Soil**	***p* Value**
TN	88.01 ± 9.51	55.67 ± 7.84	0.0001
TOC	1064.83 ± 80.90	753.33 ± 118.21	0.0003
NH4	10.03 ± 4.64	3.20 ± 2.40	0.0009
NO3	89.70 ± 28.80	22.15 ± 6.03	0.0001
Resp	278.70 ± 51.35	237.52 ± 75.58	0.2952
pH	6.05 ± 0.22	5.89 ± 0.19	0.207
% Sand	78.82 ± 4.29	81.62 ± 3.32	0.592
% Silt	11.48 ± 3.67	14.88 ± 3.64	0.235
% Clay	6.31 ± 1.24	6.91 ± 1.32	0.843
BD	2.4 ± 0.1	2.5 ± 0.07	0.761

**Table 2:** The results of ANOSIM and CAP assessments to determine the differences in the taxonomic community compositions of the total bacteria, bacterial pathogens, total fungi, and fungal pathogens in soils from the plant root soil and plant bulk soil of diseased and healthy *Ocotea monteverdensis* planted in a Costa Rican primary forest for 1 year.

ANOSIM Results	Total Bacteria		Bacterial Pathogens		Total Fungi		Fungal Pathogens	
Global test results	R = 0.373, *p* = 0.0002		R = 0.366, *p* = 0.0003		R = 0.347, *p* = 0.002		R = 0.323, *p* = 0.0001	
Pairwise comparisons	R Stat	*p* Value	R Stat	*p* Value	R Stat	*p* Value	R Stat	*p* Value
Healthy vs diseased plant root soils	0.566	0.003	0.563	0.004	0.752	0.004	0.554	0.002
Healthy vs diseased plant bulk soils	0.419	0.0002	0.485	0.002	0.046	0.273	0.231	0.05
**CAP Results**	**Total Bacteria**		**Bacterial Pathogens**		**Total Fungi**		**Fungal Pathogens**	
Comparisons	CAP R^2^	*p* Value	CAP R^2^	*p* Value	CAP R^2^	*p* Value	CAP R^2^	*p* Value
Healthy vs diseased plant root soils	0.788	0.0001	0.876	0.0002	0.801	0.0022	0.855	0.0008
Healthy vs diseased plant bulk soils	0.706	0.0001	0.723	0.0002	0.223	0.0022	0.599	0.008

**Table 3:** Results of the richness and diversity analyses for the total bacteria, bacterial pathogens, total fungi, and fungal pathogens (identified as described in the Section 2.3) in the plant root soils and plant bulk soils of diseased and healthy *Ocotea monteverdensis* planted in a Costa Rican primary forest for 1 year.

	Total Bacteria		Bacterial Pathogens		Total Fungi		Fungal Pathogens	
Diversity Index Comparisons	Richness	Diversity	Richness	Diversity	Richness	Diversity	Richness	Diversity
Healthy plant root soils	330 ± 113	5.64 ± 0.28	14.50 ± 3.79	2.51 ± 0.23	23.51 ± 23.52	2.74 ± 0.71	8.51 ± 7.58	1.76 ± 0.80
Diseased plant root soils	335 ± 129	5.54 ± 0.43	14.15 ± 4.03	2.54 ± 0.24	86.83 ± 19.56	4.26 ± 0.26	20.17 ± 3.87	2.77 ± 0.21
Mann–Whitney *p* value	0.468	0.639	0.871	0.774	0.0002	0.0003	0.004	0.005
Healthy plant bulk soil	322 ± 57	5.67 ± 0.16	14.33 ± 2.58	2.58 ± 0.17	42.67 ± 24.29	3.44 ± 0.66	15.50 ± 7.69	2.44 ± 0.61
Diseased plant bulk soil	308 ± 132	5.51 ± 0.42	14.57 ± 4.40	2.55 ± 0.29	59.67 ± 36.39	3.72 ± 0.73	13.17 ± 5.60	2.40 ± 0.43
Mann–Whitney *p* value	0.813	0.404	0.911	0.827	0.262	0.502	0.571	0.898

**Table 4:** The mean proportion of DNA sequences (MPS) of the bacterial genera with MPS values > 1% in the plant root soils and plant bulk soils of diseased and healthy *Ocotea monteverdensis* planted in a Costa Rican primary forest for 1 year. Statistically different mean values (all *p* values < 0.03) are indicated by an *.

		% MPS Diseased	% MPS Healthy	M-W
Bacterial Taxa	Function	Plant Bulk Soil	Plant Bulk Soil	*p* Value
*Clostridium*	CCD, N-Fix, AMO, possible plant pathogen	3.50 ± 2.82	0.74 ± 0.78	0.001 *
*Udaeobacter*	Probable CCD	3.45 ± 1.62	6.19 ± 1.98	0.005 *
*Gemmata*	Probable CCD	3.42 ± 0.87	4.80 ±1.77	0.029 *
*Pseudolabrys*	CCD	3.22 ± 1.54	0.94 ± 0.92	0.003 *
*Solibacter*	CCD	3.20 ± 1.74	6.52 ± 3.40	0.105
*Pedomicrobium*	CCD	3.09 ± 1.15	0.68 ± 0.20	0.001 *
*Vicinamibacter*	CCD	2.65 ± 0.87	1.27 ± 1.10	0.015 *
*Bradyrhizobium*	CCD, N-Fix, AMO	2.37 ± 0.55	2.28 ± 0.83	0.355
*Bacillus*	CCD, N-Fix, AMO, possible plant pathogen	1.84 ± 0.94	2.73 ± 1.21	0.015 *
*Methyloligella*	Methylotrophs	1.81 ± 0.73	0.56 ± 0.60	0.004 *
*Rokubacteriales*	Unclear	1.70 ± 0.67	0.44 ± 0.46	0.002 *
*Blastocatellia*	CCD	1.55 ± 1.20	0.22 ± 0.08	0.001 *
Acidobacteria (subgroup 2)	CCD	1.51 ±2.35	6.38 ± 3.45	0.003 *
*Mycobacterium*	CCD	1.51 ± 0.56	0.42 ± 0.29	0.001 *
*Gaiella*	CCD	1.45 ± 0.67	0.48 ± 0.76	0.015 *
*Nitrospira*	AMO	1.45 ± 0.66	1.15 ± 0.36	0.105
*Pirellula*	AMO	1.28 ± 0.54	0.33 ± 0.51	0.002 *
*Reyranella*	Unclear	1.22 ± 0.53	0.40 ± 0.38	0.003 *
*Actinobacteria*	CCD	1.13 ± 0.54	0.11 ± 0.17	0.001 *
*Xiphinematobacter*	Unclear	1.11 ± 0.92	3.05 ± 1.30	0.001 *
*Solirubrobacter*	Unclear	1.10 ± 0.49	0.17 ± 0.29	0.001 *
		**% MPS Diseased**	**% MPS Healthy**	**M-W**
**Bacterial Taxa**	**Function**	**Plant Root Soil**	**Plant Root Soil**	***p* Value**
*Xanthobacter*	CCD	8.03 ± 4.10	6.77 ± 0.57	0.628
*Flavobacterium*	CCD	6.80 ± 13.04	4.67 ± 1.78	0.138
*Udaeobacter*	Probable CCD	4.76 ± 3.19	4.01 ±1.81	1
*Solibacter*	CCD	4.65 ± 3.88	1.86 ± 0.36	0.731
Acidobacteria (Subgroup 2)	CCD	4.26 ± 3.72	1.79 ± 0.83	0.534
*Gemmata*	Probable CCD	3.27 ± 2.32	3.66 ± 1.32	1
*Clostridium*	CCD, N-Fix, AMO, plant pathogen	3.18 ± 2.82	0.36 ± 0.38	0.019 *
*Bacillus*	CCD, N-Fix, AMO, plant pathogen	2.73 ± 0.61	2.31 ± 1.27	0.945
*Acidobacteria*	CCD	2.64 ± 1.83	2.08± 0.40	0.628
*Rhizobium*	CCD, N-Fix, AMO	2.39 ± 2.72	0.0.33 ± 0.07	0.731
*Bradyrhizobium*	CCD, N-Fix, AMO	2.31 ± 0.65	2.86 ± 0.61	0.181
*Xiphinematobacter*	Unclear	2.89 ± 2.00	1.59 ± 0.57	0.775
*Ktedonobacter*	Unclear	1.94 ± 1.78	0.28 ± 0.18	0.153
*Elsterales*	Unclear	1.81 ± 1.80	0.74 ± 0.75	0.836
*Bryobacter*	CCD	1.79 ± 1.24	0.87 ± 0.23	0.253
*Acidibacter*	Unclear	1.33 ± 0.65	0.83 ± 0.31	0.138
*Acidothermus*	CCD	1.18 ± 0.72	1.00 ± 0.26	0.295
*Nitrospira*	AMO	1.18 ± 0.45	1.00 ± 0.27	0.181
*Burkholderia*	CCD, N-Fix, AMO, plant pathogen	1.11 ± 0.92	3.05 ± 1.30	0.008 *

**Table 5:** The mean proportion of DNA sequences (MPS) of the fungal genera with MPS values > 1% in the plant root soils and plant bulk soils of diseased and healthy *Ocotea monteverdensis* planted in a Costa Rican primary forest for 1 year. Statistically different mean values (all *p* values < 0.05) are indicated by an *.

		% MPS Diseased	% MPS Healthy	M-W
Fungal Taxa	Function	Plant Bulk Soil	Plant Bulk Soil	*p* Value
*Apiotrichum*	CCD	45.28 ± 29.11	48.26 ± 29.96	0.3367
*Rozella*	Fungal, Oomycete, algae parasite	4.17 ± 6.96	0.88 ± 0.91	0.259
*Mycosphaerella*	Plant pathogen	3.71 ± 2.82	4.93 ± 4.32	0.7478
*Saitozyma*	CCD	10.09 ± 26.88	3.15 ± 4.79	0.0782
*Glomus*	ARM	1.54 ± 3.13	2.88 ± 3.71	0.1488
*Dactylonectria*	Plant pathogen	3.77 ± 8.39	0.01 ± 0.02	0.4418
*Ilyonectria*	Plant pathogen	1.28 ± 0.84	8.68 ± 5.16	0.0039 *
*Monochaetia*	Plant pathogen	1.03 ± 2.52	0.02 ± 0.01	0.3173
		**% MPS Diseased**	**% MPS Healthy**	**M-W**
**Fungal Taxa**	**Function**	**Plant Root Soil**	**Plant Root Soil**	***p* Value**
*Apiotrichum*	CCD	26.58 ± 9.52	62.07 ± 19.02	0.0163 *
*Rozella*	Fungal, Oomycete, algae parasite	13.30 ± 7.22	0.65 ± 0.75	0.0038 *
*Mycosphaerella*	Plant pathogen	11.65 ± 4.64	3.20 ± 1.84	0.0039 *
*Archaeorhizomyces*	Plant root-associated	11.26 ± 6.54	0.23 ± 0.50	0.0033 *
*Saitozyma*	CCD	5.17 ± 5.03	9.74 ± 5.00	0.0781
*Dipodascus*	Plant pathogen	2.76 ± 4.66	0.01 ± 0.01	0.0021 *
*Didymella*	Plant pathogen	2.37 ± 2.25	0.09 ± 0.21	0.0028 *
*Acrocalymma*	Plant pathogen	1.97 ± 1.59	0.37 ± 0.66	0.0225 *
*Glomus*	ARM	1.48 ± 1.16	3.00 ± 1.89	0.1093
*Tremella*	CCD, fungal parasite	1.01 ± 1.54	0.06 ± 0.15	0.0493 *

## Data Availability

All DNA sequencing data were submitted to the NCBI-Sequence Read Archive (SRA) repository with SRA Project Code PRJNA1019742 and PRJNA646467. All other data are available upon request.

## Supplementary Materials

The following supporting information can be downloaded at: https://www.mdpi.com/article/10.3390/microorganisms13071682/s1, Table S1: Sources for identification of potential fungal and bacterial pathogens and functional groups.
