# Supplementary material for: Initial Analysis of Plant Soil for Evidence of Pathogens Associated with a Disease of Seedling Ocotea monteverdensis
Source: Microorganisms. 2025 Jul 17;13(7):1682. doi: 10.3390/microorganisms13071682 (PMC12300691; doi:10.3390/microorganisms13071682)
Supplement: Supplementary file 1 [file microorganisms-13-01682-s001.zip › microorganisms-3760644-supplementary.pdf]

Table S1. Sources for identification of potential fungal and bacterial pathogens and functional groups.

| |
|---|
| 1. Agrios, G.N. 2005. *Plant Pathology, 5$^{th}$ Edition*, Academic Press, London, UK, 2005. |
| 2. FAPROTAX . Accessed November, 2024. (https://pages.uoregon.edu/slouca/LoucaLab/archive/FAPROTAX/lib/php/index.php) |
| 3. The National Collection of Plant Pathogenic Bacteria in the UK. Accessed November, 2024 (https://www.ukbrcn.org/who-we-are/national-collection-of-plant-pathogenic-bacteria-ncppb/). |
| 4. The USA National Fungus Collection (https://www.ars.usda.gov/) |
| 5. Põlme, S., Abarenkov, K., Henrik, N.R., Lindah, B.D., et al. FungalTraits: a user-friendly traits database of fungi and funguslike stramenopiles. Fungal Divers. 2020, 105,1–16. |
| 6. Pathogens causing plant diseases by the University of California Agriculture and Natural Resources. Accessed November, 2024. (https://ipm.ucanr.edu/PMG/diseases/diseaseslist.html) |
| 7. Fungal plant pathogens list collated in Wikipedia. Accessed November, 2024 (https://en.wikipedia.org/wiki/Category:Fungal_plant_pathogens_and_diseases |
